# Balancing Heterogeneous Image Quality for Improved Cross-Spectral Face Recognition

**DOI:** 10.3390/s21072322

**Published:** 2021-03-26

**Authors:** Zhicheng Cao, Xi Cen, Heng Zhao, Liaojun Pang

**Affiliations:** Molecular and Neuroimaging Engineering Research Center of Ministry of Education, School of Life Science and Technology, Xidian University, Xi’an 710071, China; zccao@xidian.edu.cn (Z.C.); xicen@stu.xidian.edu.cn (X.C.); hengzhao@mail.xidian.edu.cn (H.Z.)

**Keywords:** cross-spectral face recognition, deblurring, deep learning, denoising, infrared, quality imbalance

## Abstract

Matching infrared (IR) facial probes against a gallery of visible light faces remains a challenge, especially when combined with cross-distance due to deteriorated quality of the IR data. In this paper, we study the scenario where visible light faces are acquired at a short standoff, while IR faces are long-range data. To address the issue of quality imbalance between the heterogeneous imagery, we propose to compensate it by upgrading the lower-quality IR faces. Specifically, this is realized through cascaded face enhancement that combines an existing denoising algorithm (BM3D) with a new deep-learning-based deblurring model we propose (named SVDFace). Different IR bands, short-wave infrared (SWIR) and near-infrared (NIR), as well as different standoffs, are involved in the experiments. Results show that, in all cases, our proposed approach for quality balancing yields improved recognition performance, which is especially effective when involving SWIR images at a longer standoff. Our approach outperforms another easy and straightforward downgrading approach. The cascaded face enhancement structure is also shown to be beneficial and necessary. Finally, inspired by the singular value decomposition (SVD) theory, the proposed deblurring model of SVDFace is succinct, efficient and interpretable in structure. It is proven to be advantageous over traditional deblurring algorithms as well as state-of-the-art deep-learning-based deblurring algorithms.

## 1. Introduction

Face recognition as a research problem has been intensively studied to date, although most works have assumed a working spectrum of visible light. To break the limits of face recognition under visible light, some scholars and research teams have turned to face recognition using the infrared (IR) spectrum, which is still a growing research topic [1,2,3,4,5,6,7,8,9,10]. Cross-spectral face recognition between IR and visible light imageries takes us beyond these limits and allows recognition to be performed at nighttime or in harsh environments such as fog, haze, and rain [11,12]. Unlike face recognition in visible light, cross-spectral face recognition involves matching between heterogeneous imageries. For instance, face images collected in the IR spectrum, such as near-infrared (NIR) and short-wave infrared (SWIR) [13], are treated as probes. They are matched against a gallery of visible light images. Such a heterogeneous matching problem is fairly challenging since the imaging mechanism and characteristics underlying IR are quite distinct from those underlying visible light. Despite such a huge difference between the heterogeneous imageries, scholars have developed tools that can extract common features shared by IR and visible-light facial images, and the technique of cross-spectral face recognition has witnessed some successes to a certain extent [3,14].

The problem of cross-spectral face recognition encounters a number of challenging issues. A lot of attention has been paid to the issue of designing powerful operators and classifiers for common features across the spectrum, such as in the work of Jain et al., who performed LDA reduction on LBP and HOG features of heterogeneous IR and visible light faces [15]. The work of Savvides et al. used a joint dictionary learning and reconstruction for NIR-VIS face recognition [4]. Some other scholars have studied the issue of facial occlusion and periocular recognition in the context of cross-spectral face recognition, such as Schmid [14,16]. As the technique of deep learning emerges and develops, scholars have recently turned their attention to applying deep neural networks for more robust feature extraction [17,18,19]. There are also scholars trying to transform the cross-spectral recognition problem into the intra-spectral recognition problem by heterogeneous conversion between IR and visible light faces, such as shown in the works of [20,21].

**Figure 1:**
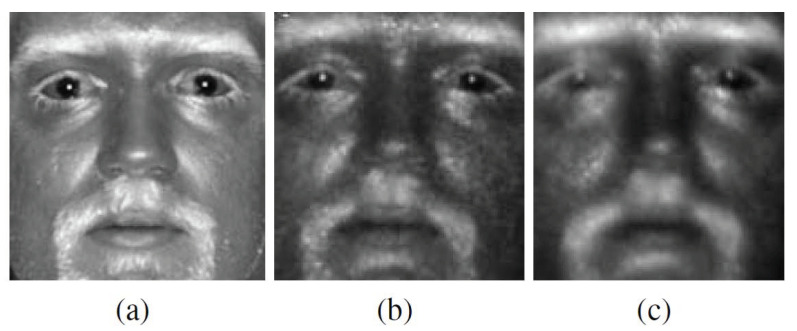
Illustrated is the phenomenon of quality degradation of IR facial images as the acquisition standoff increases. From left to right, images of the same individual collected at (**a**) 1.5 m, (**b**) 50 m and (**c**) 106 m are shown. Picture source: [22], copyright by the SPIE and reprinted with permission.

However, so far, very little research work has been conducted to particularly deal with the difference in image quality of heterogeneous faces, i.e., the issue of heterogeneous quality imbalance [22]. Such an issue cannot be overlooked, since it plays an important role in a practical recognition system in which the performance is often greatly effected by image quality [23,24]. The issue becomes even more serious when the standoffs of the heterogeneous faces are different [25]. For example, visible face images acquired in the daytime usually have better quality than IR face images collected at nighttime. As the standoff distance increases, the quality of IR images gets worse and the issue of heterogeneous quality imbalance becomes more aggravated. Such a phenomenon is illustrated in Figure 1. In view of such a problem, this research work is thus dedicated to the issue of heterogeneous quality imbalance in the context of cross-spectral face recognition and proposes a set of tools to mitigate this issue.

Common reasons accounting for degraded face images are poor lighting, off-angle, occlusion, defocus blur, camera noise, atmospheric conditions (fog, snow, rain, etc.), and other external or internal factors [26,27,28]. Sharpness, one of the most significant factors of image quality, is crucial to the performance of a face recognition system. The majority of face recognition algorithms extract both geometric information as well as refined textural features. Both require a sharp and clear face image [24]. Other factors, such as contrast, brightness and illumination, also play important roles in face recognition. However, in IR images, their effect is not as pronounced as that of image sharpness. Therefore, we focus on balancing the sharpness of heterogeneous faces in this work.

In order to achieve the goal of quality balancing, one can address the problem from either the obverse side or the reverse side: (1) out of two matched faces, to bring the face of higher quality (i.e, the visible light face in our study) down to the level of quality of the other face (i.e., the IR face); (2) to improve the face of lower quality to the level of quality of the other face. The first approach is conceptually more direct and easier to realize in practice, such as by simply smoothing the face image of higher quality with a low-pass kernel. The second approach is obviously more complex and can be accomplished via image enhancement such as denoising. We in this paper take the second type of approach. However, denoising alone typically results in removal of useful facial details. Hence, to overcome this drawback, we further propose combining denoising with a subsequent stage of deblurring. More specifically, we first denoise the IR face images with the BM3D algorithm and then deblur them with a new CNN-based algorithm that we propose.

To summarize, this paper addresses the issue of quality imbalance in the context of cross-spectral face recognition and claims the following contributions.

- To the best of our knowledge, this is one of the first works that specifically deal with the issue of quality imbalance in cross-spectral face recognition.
- Quality balancing is achieved by upgrading the IR face imagery where a cascaded structure of denoising and deblurring is proposed.
- For deblurring, we further propose an SVD theory-inspired CNN model (SVDFace) which decomposes the inverse kernel function into stacks of 1D convolutional layers. The singular value decomposition (SVD) network has advantages such as compact parameters, good interpretability in its structure, and needless knowledge of the exact cause of image degradation.
- The proposed deblurring method (SVDFace) is proven to be advantageous over other deblurring methods, including state of the art. The cascaded structure of face enhancement is also shown to be superior to the non-cascaded structure. Moreover, the upgrading approach to quality balancing outperforms the downgrading approach.

The remainder of the paper is organized as follows. In Section 2, we review related research works on the topic of image quality imbalance as well as on the topic of deblurring. Section 3 introduces the proposed methodology for balancing the heterogeneous image quality, where the cascaded enhancement structure is described and the SVD-inspired deblurring network is proposed and explained. In Section 4, we describe the datasets used in our experiments and the experimental setup. We also analyze and compare the recognition performance of our method with other methods from different perspectives. Section 5 summarizes the work.

## 2. Related Works

To date, the literature contains very scarce research work on the topic of heterogeneous quality imbalance in the context of cross-spectral face recognition. There are, nonetheless, a considerable number of research studies on a broader scope of image quality for general biometrics.

For example, Grother et al. studied the issue of fingerprint image quality in their paper [29], in which they found that the image quality of the fingerprints greatly affected the matching performance of their fingerprint matcher. Using a measure of the Normalized Matching Score (NMS) proposed by them, it was demonstrated that the quality of the fingerprint samples is related to NMS, especially for the genuine scores. The NIST fingerprint dataset was utilized in their experiment.

Nandakumar et al. took an approach of statistical tests to address the quality problem. They estimated the joint densities of quality and matching scores for both genuine and imposter distributions. Then, they conducted a likelihood ratio test between the estimated genuine and imposter distribution. The method was demonstrated on fingerprint and iris biometrics [30]. For each modality, a quality-based density was evaluated and a multi-modal distribution was obtained as a product of the individual density modalities. Experimental results showed that it indeed improved the verification performance when combining the modalities with the quality measures.

In the work of [31], Jain et al. modeled the distributions of genuine and impostor match scores as finite Gaussian mixture models, of which the parameters were estimated with the EM algorithm. They proposed a framework for the optimal combination of match scores that was based on a likelihood ratio test. In addition to handling the quality issue, their method could also address other issues such as discrete values, arbitrary scales, and correlation between the scores of multiple matchers. The proposed fusion framework was proven to be successful by experiments on three multi-biometric databases.

Kryszczuk and Drygajlo [32,33] proposed using the quality measures and matching scores in order to improve the accuracy of uni- and multi-modal biometric classification. They introduced a stacking-based classifier ensemble (named Q-stack), which concatenated matching scores of the original matcher and quality measures at the matching stage. The method was shown to be useful for tasks of biometric identity verification using face and fingerprint modalities. Classifiers such as SVM, Bayes classifiers and Linear Discriminant were tested.

In summary, all of the aforementioned works either assess the image quality and define a certain measure (or score) to accept/discard a biometric sample or to use the quality information as a weak feature for the recognition task. However, improving low-quality IR images and compensating the quality imbalance remain unstudied.

We in this paper study image quality from a different perspective—to balance heterogeneous image quality, and we propose addressing the heterogeneous quality imbalance problem by a CNN-based deblurring method preceded by denoising of the low-quality IR faces. Therefore, we hereby also give a review of image deblurring.

Image deblurring is usually formulated as a deconvolution problem, which has been intensively studied. However, the majority of previous works tackle the problem from a generative perspective assuming a known image noise model and known distributions that natural image gradients follow. For example, in the work by Richardson [34], describing the Richardson-Lucy method, image noise is assumed to follow a Poisson distribution.

Whyte et al. [35] introduced an algorithm that locates and decouples error-prone bright pixels for deblurring shaken and partially saturated images. In their work [36], Kenig et al. added an explicit denoising module to the deconvolution module, where the denoising approach is trained from noisy data. The generative approaches typically have difficulties to handle complex outliers that are not independent and identically distributed.

More recently, deep neural networks have been successfully applied to many image processing tasks. Such efforts become possible due to the availability of a large number data and enormous computational resources available to us today. Very promising results with respect to conventional methods have been achieved and displayed [37]. There are some works where deep learning has been applied to deblurring [38], such as the works of [39,40,41,42]. In this paper, we propose the use of deep neural networks for automatic learning of the deconvolution operations (i.e., deblurring) without the need to know the exact cause of image blurring and degradation.

## 3. Proposed Methodology

The methodology proposed in this paper for quality balancing between the heterogeneous imagery is to upgrade the quality of IR images by means of image enhancement. Specifically, we achieve image enhancement with a cascaded structure of denoising and deblurring.

### 3.1. Necessity of the Cascaded Structure

Denoising of low-quality IR faces can improve image quality and compensate the heterogeneous quality imbalance to a certain extent. However, we find that a combination of denoising and deblurring is still necessary. The reason we propose such a combination is that there exists a paradox in denoising of IR faces: denoising alone is known to simultaneously remove useful facial details while suppressing unwanted noise.

**Figure 2:**
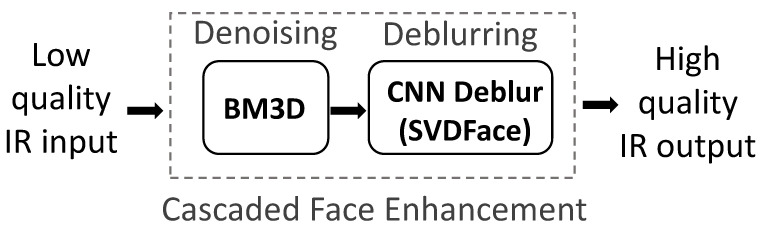
The overall structure of the cascaded face enhancement approach proposed in this work, which involves consecutive denoising and deblurring.

These facial details, however, usually contain information crucial to face recognition. As a result, with the level of noise coming down, the denoised face images yield higher-quality metrics such as signal-to-noise ratio (SNR). However, they ultimately demonstrate bad recognition performance. Since our final goal is to improve the cross-spectral recognition performance, we want to recover the refined facial details as much as possible while maintaining a low noise level. This motivates us to add an additional block in the image enhancement block diagram (see Figure 2): a successive deblurring block after the preceding denoising block. The two-stage enhancement structure is therefore ensured to have the advantage of retaining the useful facial details while suppressing the noise.

For denoising, we utilize an existing well-known technique called BM3D [43], which is based on sparse representation in a 3D transform-domain. BM3D has been proven to be successful and robust in various denoising tasks, which is why it is chosen as the denoising module in our paper. With the denoising stage being set, the remaining stage of deblurring is the tricky one left, which is the core of our proposed cascaded approach. As for deblurring, we especially propose a new deep neural-network-based algorithm due to the fact that traditional deblurring methods either presume knowledge of the image degradation cause or demonstrate low robustness. With the advent of deep learning techniques, the deblurring problem can be addressed by convolution neural networks. After a thorough analysis of the deblurring problem (refer to Section 3.3.1), we observe that the deblurring process can be modeled and learned as convolutional operations of CNN without the need to know the exact cause of image blurring and degradation.

### 3.2. BM3D Denoising

The image denoising algorithm of BM3D is an adaptive non-parametric filtering approach, which uses a strategy based on enhanced sparse representation in a transformed domain. The central idea behind it is to group similar 2D image blocks into 3D data arrays followed by collaborative filtering. The denoised output is eventually a 3D estimation that consists of the jointly filtered grouped image blocks. Since the way to develop BM3D denoising is not the focus of this work and a detailed explanation of the BM3D algorithm can be found in [43], we hereby only provide a concise depiction.

When applied as the denoising module in this paper, we first generate two-dimensional image blocks out of a low-quality-input IR face, according to the similarity of the IR face blocks. Stacking up similar image blocks, a 3D array is constructed afterwards, which is called an image group. During aggregation of blocks, we use the same block-matching method that has been used in motion estimation for video compression. Following grouping, the stage of collaborative filtering is conducted, which produces estimates of the ideal non-noisy image in a way such that each group of blocks collaborates for the filtering of all other groups and vice versa. Specifically, the collaborative filtering is realized as shrinkage in a 3D domain, which comprises three steps. Firstly, a 3D linear transformation is performed on the image groups; secondly, the transform coefficients are shrunken to attenuate the image noise via soft/hard thresholding or Wiener filtering; lastly, inverse 3D transform is performed to produce estimates of all grouped image blocks. During aggregation, the basic estimate of the ground truth image is computed by weighted averaging of all the obtained block-wise estimates that are overlapping. A final estimate step is also added for better denoising performance, where an improved grouping and collaborative Wiener filtering is involved.

**Figure 3:**
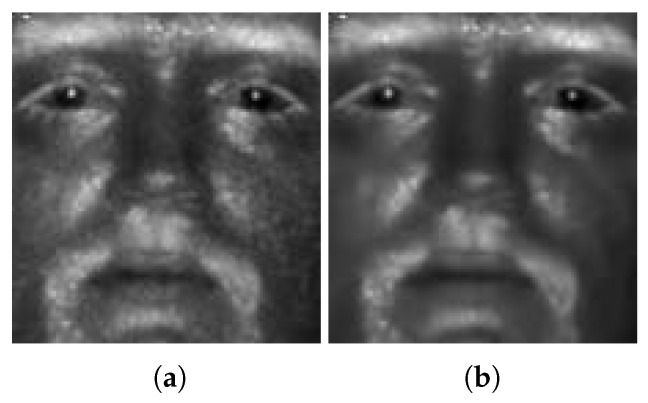
Results of BM3D denoising: (**a**) the original SWIR 50 m face image and (**b**) the same image after denoising by means of BM3D. Note that BM3D not only suppresses the noise but also removes useful facial details, resulting in a blurry face image.

The result of applying the BM3D denoising algorithm to an SWIR face image is displayed in Figure 3b. Compared to Figure 3a, the original noisy input, one can easily see that the quality of the SWIR image is improved (based on visual evaluation). It is worth noting that ideal denoising should yield a clean and sharp output. However, BM3D denoising, just like other denoising techniques, not only suppresses the noise but also removes useful facial details, resulting in a blurred face image. This leads to our cascaded structure with a subsequent deblurring stage, as proposed in the next subsection of Section 3.3.

### 3.3. Deep Neural-Network-Based Deblurring

BM3D removes camera noise and image distortions due to atmospheric interference. However, it also decreases the quality of fine features in the face image that are useful for automatic face recognition. In the Fourier transform space, fine features are described by high frequency components and thus denoising, which is a low-pass operation, may oversmoothe the fine features. To enhance the facial details, we propose applying a deblurring stage right after BM3D denoising. The process of image blurring can be modeled as a translation-invariant convolution operation. Thus, to restore the fine features in the face image, we have to perform deconvolution.

#### 3.3.1. SVD-Inspired Deblurring Network

Image deblurring is a well-known problem in the area of image processing and has been intensively studied to date. Traditionally, deblurring is achieved by solving the inverse of the distortion cause. The problem is, however, that more than often, the actual distortion function is unknown and difficult to formulate. As techniques of deep learning appear, the inverse problem can be learned and approximated in an implicit way. Nonetheless, success of these deep learning methods relies on large training data, which are not available in our specific case of cross-spectral face recognition. IR face data are relatively expensive and difficult to collect compared to visible light data.

In view of this, we propose to learn the inverse problem with a more succinct and efficient neural network. Inspired by the theory of singular value decomposition (SVD), we find that ordinary 2D convolution operations of the neural networks can be replaced by 1D operations. Such an improvement is supported by the SVD analysis of the inverse problem in deblurring. Consequently, the new network has high interpretability, which is usually not the case for other ordinary networks. More importantly, the SVD-inspired network has much fewer parameters than other ordinary networks, an advantage especially crucial to our specific topic.

To formally present our SVD-inspired networks, we start with the mathematical formulation of the image deblurring problem (i.e., deconvolution). The central idea behind deconvolution is to reverse the process of image distortion, which is defined by a kernel function called the Point Spread Function (PSF):

(1) $$\tilde{I}=I**\;K,$$

where *I* is the ideal clean face image, *K* is the kernel function (i.e., PSF), $\tilde{I}$ is the blurry image, and ∗∗ stands for two-dimensional convolution. To deblur the blurry image, one needs to find the inverse of the kernel function, $K^{-1}$. Due to their nature, convolutional neural networks can be used to approximate this deconvolution operation. We will carefully prove this argument below. According to the separability rule, the inverse kernel can be decomposed using singular value decomposition (SVD):

(2) $$K^{-1}=U\Sigma V^{T},$$

where U and V are unitary matrices and Σ is a rectangular diagonal matrix (i.e., all entries rather than $\Sigma_{ii}$ are zero).

Using block matrix multiplication, the SVD equation can be further expanded as

(3) $$\begin{array}{cc} K^{-1} & =U_{p\times m}\Sigma_{m\times n}V_{n\times q}^{T}\\ \; & =\left[\begin{array}{c} u_{1},u_{2},\cdots ,u_{m} \end{array}\right]\left[\begin{array}{cccccc} \sigma_{1} & \; & & \cdots & \cdots & 0\\ \; & \sigma_{2} & \; & & \; & \vdots \\ \vdots & \; & \ddots & \; & & \\ 0 & \; & & \sigma_{m} & \cdots & 0 \end{array}\right]\left[\begin{array}{c} v_{1}^{T}\\ v_{2}^{T}\\ \vdots \\ v_{n}^{T} \end{array}\right]\\ \; & =\sum_{i=1}^{m}\sigma_{i}u_{i}v_{i}^{T}, \end{array}$$

where $u_{i}$ and $v_{i}$ are the *i*-th column of the unitary matrices U and V, respectively, and $\sigma_{i}$ is the *i*-th singular value of Σ.

**Figure 4:**
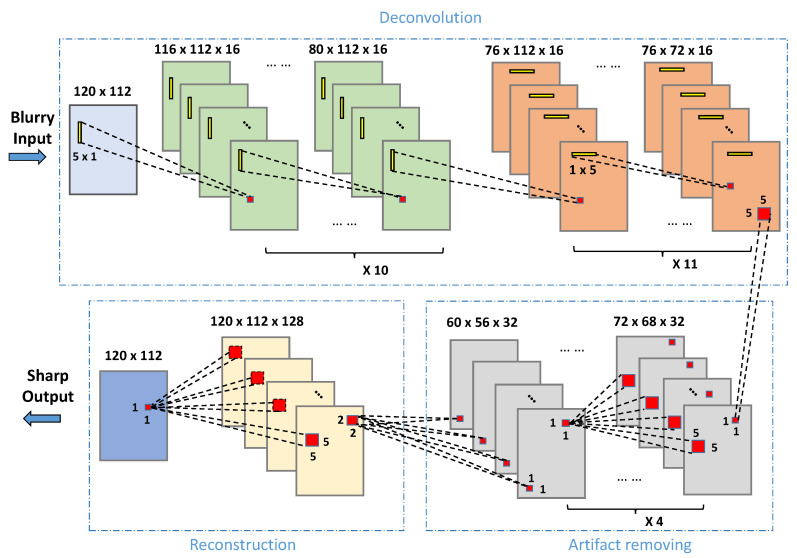
The framework of the proposed CNN-based deblurring algorithm, which consists of deconvolution, artifact removing and reconstruction.

Thus, the deconvolution process can be rewritten as:

(4) $$\begin{array}{cc} \hat{I} & =\tilde{I}**\;K^{-1}\\ \; & =\sum_{i=1}^{m}\sigma_{i}\;\tilde{I}*u_{i}*v_{i}^{T}, \end{array}$$

where $\hat{I}$ is the deblurred output and * stands for one-dimensional convolution. Equation (4) shows that due to the fact that the product $u_{i}v_{i}^{T}$ can be viewed as a two-dimensional separable function, a two-dimensional convolution can be represented as a sequence of two one-dimensional convolutions. With regard to the composition of the convolutional neural network for deblurring, this conclusion suggests that we need to design network layers with 1D convolutional kernels rather than 2D square kernels, which were traditionally used in CNN structures (see Figure 4 for details).

The next task is to determine the size of the 1D kernels. In practice, we can approximate the inverse kernel $K^{-1}$ with an image of a smaller size than the original image by disregarding near-zero values of $K^{-1}$. Empirically, choosing the size of $K^{-1}$ to be no less than 1/3 of the original image size has been shown to be a good practice. For example, the kernel size can be set as 45×41 for an input face of 120×112. However, 45×41 is still a relatively large image. In accordance with guidelines on building an effective CNN, the performance of a network tends to be higher as the convolutional kernels decrease in size, while the network increases in depth [44,45]. Therefore, we further decompose the convolutional kernel into a set of smaller kernels using the fact that a larger two-dimensional kernel is the convolution of smaller kernels. The mathematical description of the process is given as:

(5) $$u={\tilde{u}}^{\left(1\right)}*{\tilde{u}}^{\left(2\right)},$$

where u is the original larger 1D kernel and ${\tilde{u}}^{\left(1\right)}$ and ${\tilde{u}}^{\left(2\right)}$ are two smaller 1D kernels. This process can be repeated until we obtain the desired size of kernels. In our paper, we decompose the vertical 1D kernel with the original size of 45×1 into a sequence of repeated convolutions among 11 smaller kernels of the size of 5×1. Similarly, we represent the horizontal 1D kernel with the size of 1×41 as a sequence of repeated convolutions among 10 kernels with the size of 1×5.

Overall, the proposed deep neural network comprises three parts: a deconvolution module, an artifact-removing module and a reconstruction module. In the deconvolution module, the input layer takes in an IR face image of size 120×112 and is connected to two sets of convolutional layers, where the convolutional kernels of the first set are horizontal 1D filters (corresponding to $u_{i}$) and the kernels of the second set are vertical 1D filters (corresponding to $v_{i}^{T}$). The sizes of the kernels are 5×1 and 1×5, resulting in feature maps of size 76×112 and of size 76×72 at the last layer of each set, respectively.

The deconvolution module is further connected with the artifact removing module to remove outliers. The second module has four layers, C3_1 ∼ C3_4. Each of the four layers is composed of 32 kernels with the same size of 5×5. The output of the artifact removing module has the size of 60×56×32. The last module is built to reconstruct the final deblurred image, which should be reduced to the same size as that of the input to the network, i.e., 120×112. To achieve this, we use a backward convolution structure (also known as transposed convolution), which has two layers. The first layer, C4_1, is of size 2×2 up-sampling operation, while the second layer, C4_2, is of size 5×5 convolutional operation. Margins are padded to have a final output size of 120×112. The block-diagram of the proposed deep network is demonstrated in Figure 4, and its parameters are listed in Table 1.

**Figure 5:**
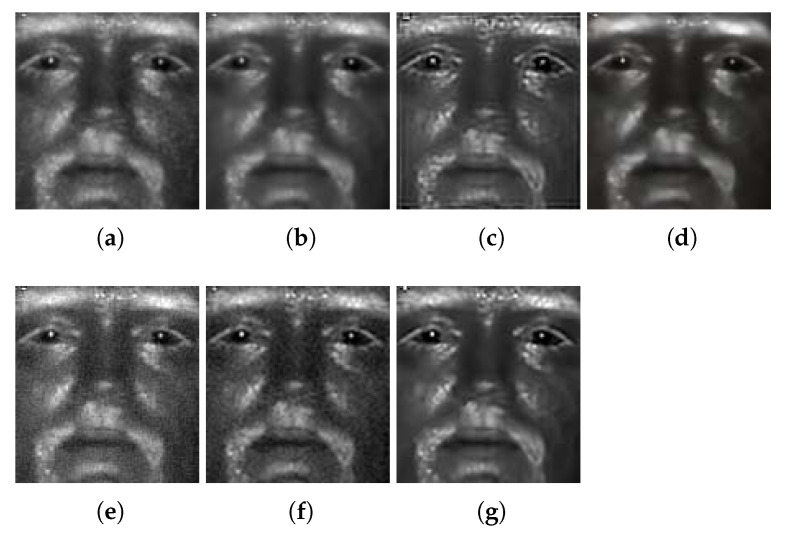
Comparison of several image enhancement methods: (**a**) Original IR face; (**b**) BM3D denoising; (**c**) BM3D denoising + blind deconvolution; (**d**) BM3D denoising + DeblurGAN; (**e**) the proposed cascaded enhancement approach. Note that (**b**) BM3D alone removes both unwanted noise and some useful facial details. (**c**) is sharper than (**b**), but artifacts are also introduced around eyelids and margins and white speckles. DeblurGAN, Progressive Semantic Deblurring and UMSN Deblurring, as shown in (**d**–**f**), all sharpen (**b**) to some extent. Overall, the proposed cascaded enhancement approach with SVDFace deblurring (**g**) outperforms all the other five approaches (zoom-in recommended for better viewing).

In order to train this SVD-based deblurring network, we adopt a transfer learning method. Firstly, we train our model on a popular visible light dataset, GoPro [41]. Then we fine-tune the model using an NIR subset of Q-FIRE [46], a multispectral dataset collected by Clarkson University. The NIR subset is composed of 1030 images from 82 subjects. We partition the NIR dataset into two parts: the first 800 images are used as training and the remaining 230 images serve as validation. Training requires pairs of images consisting of high-quality IR face images and their low-quality IR counterparts. We simulate low-quality IR face images by blurring the original face with a simple smoothing filter of size 3×3. To mimic image degradation due to camera and atmospheric effects, we add realizations of a white noise to the blurred face images. The noise level is set to σ=5. A quadratic loss function and the Adaptive Moment Estimation (Adam) rule are used during the training process. The specific model and parameters corresponding to the epoch with the best recognition performance was chosen. It should be noted that this is done in an empirical way. As an illustration, an IR face image deblurred by means of the proposed deep convolutional network is displayed in Figure 5c. Compared to the face image after denoising, as shown in Figure 5a, the final output after the deblurring stage looks much improved in its quality.

To provide a comparison to other deblurring algorithms, we also involved an implementation of blind deconvolution [47]. Given the BM3D denoised result (Figure 5b) of a low-quality IR face (Figure 5a), the result of applying blind deconvolution is shown in Figure 5c. As observed, blind deconvolution is able to successfully recover edges in the face image and makes it look sharper. However, it also introduces artifacts (ringing effects) around the eye lids and the image margins and white speckles on the face. To acknowledge the state-of-the-art techniques in image enhancement, we compare the performance of our proposed approach with the performance of DeblurGAN [42], Progressive Semantic Deblurring [48], and UMSN Deblurring [49], all of which represent the recent advances in image deblurring. As shown in Figure 5d, DeblurGAN successfully sharpens the blurry face but is unable to recover most of the useful facial details important for face recognition. When tested with our proposed model of SVDFace, the output face is clear and sharp with facial details retained (Figure 5e). This result justifies the development of the algorithm proposed in this paper.

#### 3.3.2. Analysis of Structural Advantages

Before we carry out experiments to verify the advantages of the proposed deblurring model, SVDFace, we provide a theoretical analysis of its structural advantages over ordinary neural networks. A summary of the structural advantages of the SVD-inspired model is given in Table 2.

**Figure 6:**
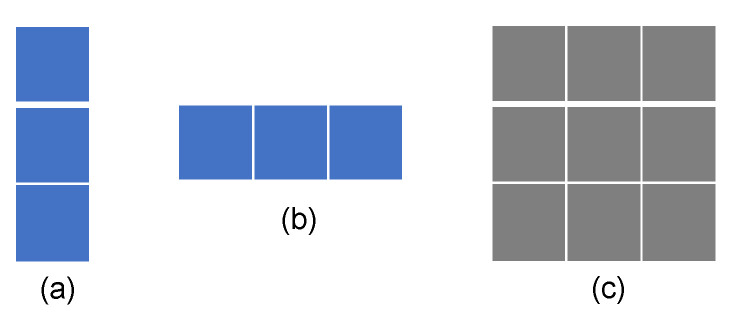
Comparison of convolution operations between singular value decomposition (SVD)-inspired network and other ordinary networks: (**a**) vertical SVD convolution; (**b**) horizontal SVD convolution; (**c**) ordinary convolution.

As can be seen in Figure 6, the most conspicuous characteristics of SVDFace is that it is in a one-dimensional shape, either vertical or horizontal, compared to the two-dimensional shape of ordinary neural networks. Such a choice of 1D shape is supported by the SVD decomposition theory applied to the inverse problem in deblurring. In other words, the new type of neural networks has higher interpretability than other ordinary networks, whose lack of interpretability has long been criticized.

Secondly, since its convolution kernel has a 1D shape, the SVD-inspired network has much fewer parameters than other 2D-shaped networks. This is an advantage especially important on the topic of cross-spectral face recognition, where the number of IR training data is usually limited. For simplicity, let us take an SVD kernel size of 1×3 for example. Assuming an SVD networks of *l* layer and each layer with *k* kernels, the total number of network parameters is 3×m×n×l×k for an input image of m×n. Such a calculation is the same for both vertical and horizontal SVD kernels. Now, if the SVD kernels are changed to ordinary 2D shaped kernels of 3×3, the total number of network parameters is 9×m×n×l×k, which is 3 times larger than that of the SVD networks. That is to say, the SVD networks can save network parameters as dramatically as 66.67%.

Furthermore, the computation and storage cost of the SVD networks is also dramatically lowered as the network parameters are reduced. This is also an advantage when designing a practical system, especially in mobile or embedded applications where computation and storage resources are very expensive. Last but not least, our SVD networks can automatically learn the inverse of PSF function in an implicit way, without any a priori knowledge of the exact cause of image blurring and degradation.

## 4. Experimental Results and Analysis

In this section, we describe several cross-spectral matching experiments and summarize the results of matching SWIR or NIR probe images of low quality to a gallery of visible light images of high quality, with or without the application of the smoothing or enhancement technique. For the SWIR and NIR cases, results are presented for both standoff distances of 50 m and 106 m.

### 4.1. Dataset

To analyze the performance of the proposed quality enhancement approaches in the context of face recognition, we involved the Tactical Imager for Night/Day Extended-Range Surveillance (TINDERS) dataset, collected by the Advanced Technologies Group, West Virginia High Tech Consortium (WVHTC) Foundation [50].

**Figure 7:**
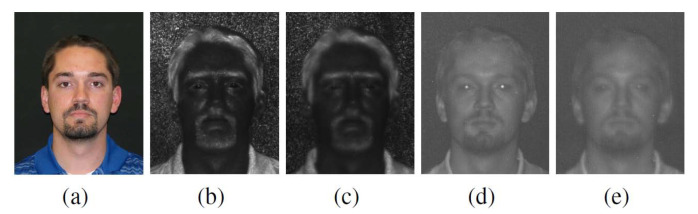
Sample images, from left to right: (**a**) visible light at 1.5 m, (**b**) short-wave infrared (SWIR) at 50 m, (**c**) SWIR at 106 m, (**d**) near infrared (NIR) at 50 m, and (**e**) NIR at 106 m.

The TINDERS dataset comprises 48 frontal face classes each represented by visible light, NIR and SWIR images. The visible light images are acquired at a short standoff distance of 1.5 m, while NIR images are collected at the wavelength of 980 nm for two long standoff distances of 50 m and 106 m, and SWIR images at the wavelength of 1550 nm are also collected at the same standoff distances. The visible light (color) images in a resolution of 480×640 are collected in two sessions, with 3 images per session. All of them have neutral expression, resulting in a total of 288 images. Visible light images are saved in the .jpg format. Within SWIR and NIR image sets, four or five images per class are available for each long-range distance. Two or three of the IR images per class have a neutral expression, and two images per class have a talking expression. A total of 478 images, each of resolution 640×512, are available in the SWIR band. A total of 489 images with a resolution of 640×512 are available in the NIR band. Both SWIR and NIR images are stored in the .png format. Sample images are shown in Figure 7.

Prior to feature extraction and matching, all images are aligned using positions of the eyes, regardless of the spectral band. Images are rotated, scaled and translated such that pairs of eyes are aligned. Color images (visible light) are converted to gray-scale images, while IR images are first processed using a logarithmic function. The transformed IR images are further normalized to have intensity values between [0,255]. During feature extraction, the heterogeneous images are encoded using a composite operator, which fuses Gabor filters, Local Binary Patterns (LBP) [51], Generalized LBP (GLBP) [52] and Weber Local Descriptor (WLD) [53]. Detailed information can be found in our previous works [14,16].

### 4.2. Quality Balancing: Upgrading vs. Downgrading

In order to justify our proposed approach of upgrading the IR faces to the problem of heterogeneous quality balancing, we design the first experiment to compare the upgrading approach against the downgrading approach. The the upgrading approach, as described in Section 3, is achieved by cascaded face enhancement, which combines the BM3D denoising algorithm and our proposed deblurring CNN model. The downgrading approach can be implemented simply through Gaussian-based smoothing of the visible light faces.

We experimented with a variety of cases in which different light spectra and standoffs are involved, namely to match IR faces of SWIR 50 m, SWIR 106 m, NIR 50 m and NIR 106 m versus visible light faces at 1.5 m. In all cases, the cross-spectral matching is conducted with and compared between three different methods: the original matching algorithm without any preprocessing of quality balancing, the downgrading approach to quality balancing via Gaussian smoothing, and the upgrading approach that we propose. Numerical analysis of the experiments is presented in the form of Genuine Accept Rate (GAR) and Equal Error Rate (EER) values. EERs and GARs of cross-spectral matching for the SWIR and NIR bands are summarized in Table 3 and Table 4, respectively.

When matching low-quality SWIR face images acquired at 50 m and 106 m to visible face images of high quality acquired at a short standoff distance of 1.5 m, as shown in Table 3, both approaches of upgrading and downgrading are beneficial for the cross-spectral face recognition performance. For the case of SWIR 50 m, the GARs at FAR =0.1 and FAR =0.001 and the EER values are 0.9188, 0.62118 and 0.0890, respectively. After Gaussian smoothing of the high-quality visible light faces, the GAR and EER values are boosted to be 0.9293, 0.6709 and 0.0792, respectively. This indicates that, easy as it may appear, the downgrading approach works well in practice. When the quality balancing approach is chosen as our upgrading approach, an even more significant improvement of performance is observed: the GAR and EER values are boosted to 0.9643, 0.6926 and 0.0553, respectively. This clearly demonstrates the superiority of our proposed upgrading approach over the easier approach of quality downgrading. As the standoff increases to 106 m, the degree of performance boost becomes higher. This suggests that the concept of quality balancing is even more helpful for cross-spectral face recognition at a longer standoff, which in turn justifies the very topic of this study.

We repeated this experiment for the cases of the NIR spectra at standoffs of 50 m and 106 m and obtained very similar observations. As can be seen in Table 4, both the upgrading and downgrading approaches again benefit from the recognition performance significantly, with our upgrading approach outperforming the downgrading approach. However, in the NIR case, the performance boost is not as pronounced as that in the SWIR case. This suggests that the quality imbalance issue is more serious in the NIR band and the need for heterogeneous quality balancing is more urgent.

### 4.3. Cascaded or Non-Cascaded

The next set of experiments were conducted intending to validate our proposed structure of cascaded face enhancement. In other words, we wanted to see that the stage of CNN deblurring that follows the preceding stage of BM3D denoising is indeed necessary for and contributes to the recognition performance. Therefore, we compared the recognition performance of cascaded face enhancement with that of BM3D denoising alone and that of no image preprocessing at all. This study wasconducted for all cases of different spectra with varying distance.

When experimenting with the SWIR band, as shown in Table 5, we observed that denoising alone (i.e., without the following deblurring stage) shows a slight drop in the recognition performance. The GAR at FAR =0.1 and the EER decreased from 91.88% and 8.90% to 88.23% and 11.21%, respectively, when the SWIR standoff was set at 50 m. In contrast, our proposed cascaded enhancement structure (i.e., BM3D + CNN deblur) experienced a boost in the GAR and EER values by 4.55% and 3.37%, respectively. This clearly proves that our proposed cascaded enhancement structure is indeed beneficial and necessary. As the standoff increased to 106 m, the GAR at FAR =0.1 and the EER experienced a larger drop and the improvement of the cascaded structure became more dramatic, which means the cascaded enhancement technique was more effective as the standoff distance increased. These observations are expected, since we know that denoising alone will remove useful facial details, although denoising does suppress the image noises. Thus, our proposal of adding a successive deblurring stage to the denoising stage is valid in terms of improving cross-spectral face recognition performance.

Next, we carried out the same experiment for the NIR band, and similar observations and results were obtained. As listed in Table 6, the GAR and EER values of our proposed cascaded enhancement structure are significantly higher than those of the denoising alone structure and the original algorithm for the case of NIR 50 m. As the standoff is set to be 106 m, the same conclusion still holds true.

### 4.4. Comparison of Different Deblurring Methods

The final set of experiments were designed to further justify the need for the development of the CNN deblurring model we propose in this paper (SVDFace). Therefore, we compare SVDFace with five other deblurring methods including traditional non-deep learning based algorithms and state-of-the-art deep learning models. The original recognition performance is also compared as the baseline, resulting in a total of seven methods. Specifically, the other five deblurring methods of comparison are a Laplacian sharpening based algorithm [22], a well-known blind deconvolution technique [47], and three state-of-the-art models: DeblurGAN [42], Progressive Semantic Deblurring [48], and UMSN Face Deblurring [49]. DeblurGAN is a recent GAN-based model that is very successful for deblurring of general images, while Progressive Semantic Deblurring and UMSN are SOTA algorithms designed especiallyfor face deblurring. It should be noted that all the deblurring methods are experimented on using the same denoising algorithm of BM3D as a preceded stage. This is to ensure a fair comparison.

**Figure 8:**
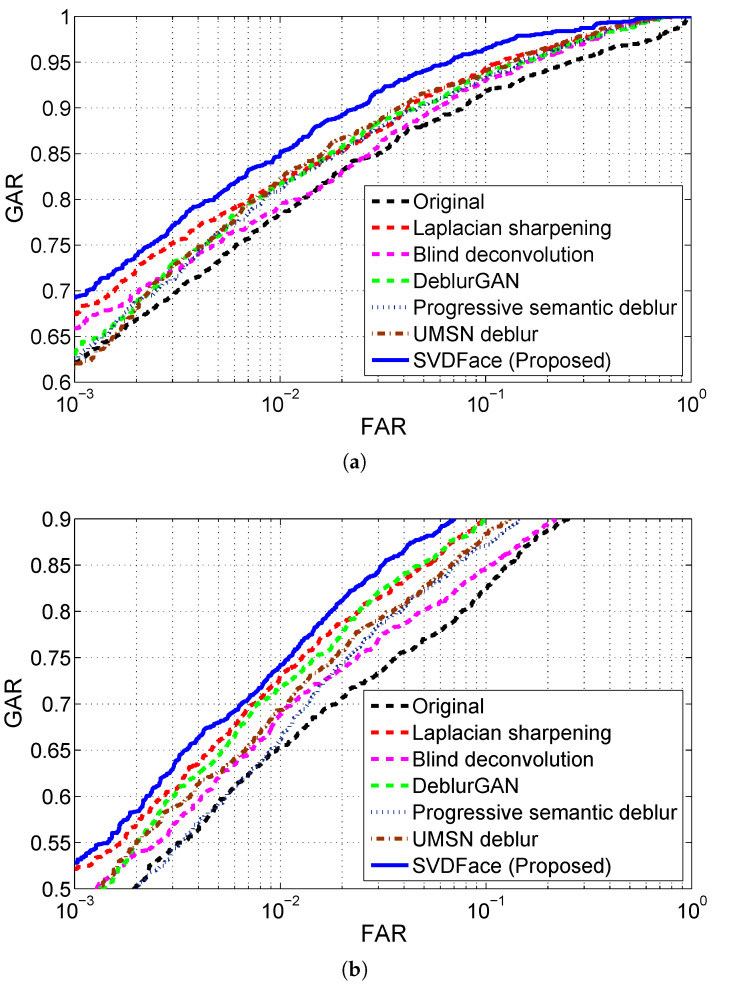
Matching SWIR probes to visible gallery using different deblurring methods: (**a**) SWIR 50 m; (**b**) SWIR 106 m.

**Figure 9:**
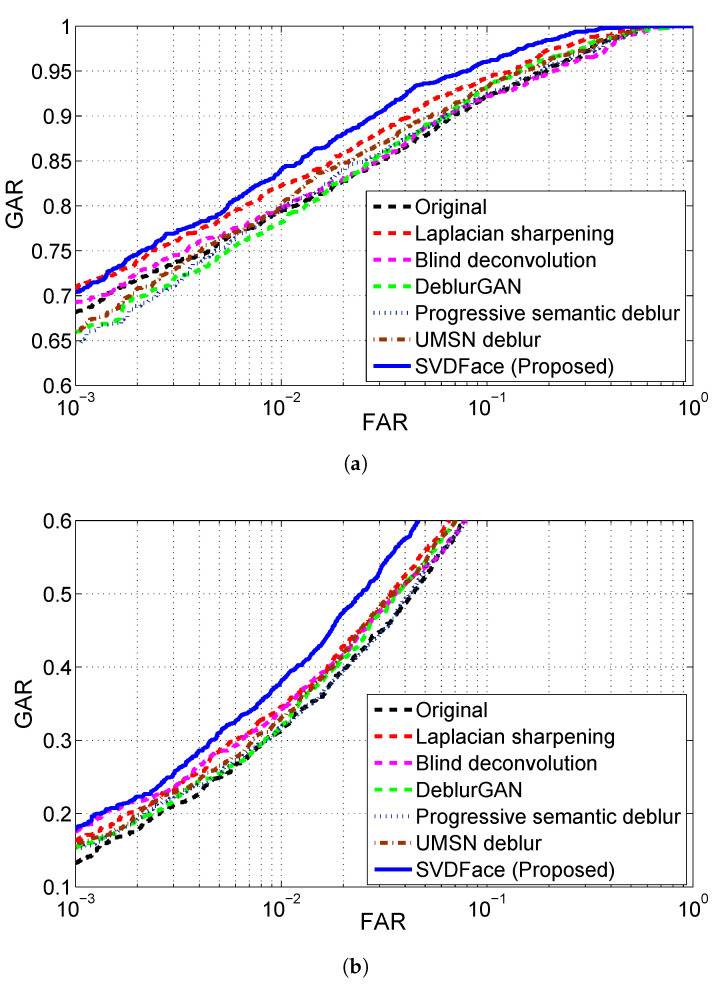
Matching NIR probes to visible gallery using different deblurring methods: (**a**) NIR 50 m; (**b**) NIR 106 m.

For all deblurring methods, IR facial images in both the SWIR and NIR bands at both 50 m and 106 m were first denoised with BM3D and then processed with the corresponding deblurring method. Finally, cross-spectral face matching of IR faces versus visible light faces was conducted, and the performance was evaluated. The experimental results of cross-spectral matching are presented in receiver operating curves (ROC) as well as GAR and EER values. The ROC curves for SWIR 50 m and 106 m are shown in Figure 8a,b, respectively. EERs and GARs are summarized in Table 7. The ROC curves for NIR are plotted in Figure 9, while EERs and GARs are listed in Table 8.

We first experimented with the SWIR spectra at varying standoffs of 50 m and 106 m. By comparing the matching performance of the proposed deblurring method of SVDFace with the performance of all other deblurring methods, we clearly see the advantage of using SVDFace—a substantial performance improvement is observed. For the case of SWIR 50 m, as shown in Table 7, our SVD-based deblurring network achieves a GAR of 96.43% at FAR =0.1 and an EER of 5.53%, in comparison to 93.56% and 7.59% of DeblurGAN, 94.05% and 7.28% of UMSN Deblurring, 93.28% and 7.85% of Progressive Semantic Deblurring, 94.33% and 7.29% of Laplacian Sharpening, and 92.99% and 8.04% of Blind Deconvolution, respectively. It should be also noted that all the four deblurring methods including ours indeed improve the recognition performance compared to the original method, which involves no quality balancing processing at all.

As the standoff increases to 106 m, the cross-spectral recognition performance drops significantly. This is attributed to the fact that IR images experience stronger noise and disturbance at a larger standoff. The good news is, however, that all four deblurring methods still work well in the longer standoff case. Once again, the proposed SVDFace takes first place and Blind Deconvolution comes last in terms of the degree of performance boost. Additionally, we find out that the degree of performance boost by our deblurring method is even larger in the longer standoff case of 106 m than that in the case of 50 m. This suggests that our proposed method of cascaded enhancement structure and the deblurring model for heterogeneous quality balancing is especially suitable for longer distance of image acquisition. In other words, our method is robust at varying image acquisition distances.

In the next experiment, NIR face images were matched against visible face images. The matching results for the standoffs of 50 m and 106 m are displayed in Figure 9a,b, respectively. EERs and GARs with FAR set to 0.1 and 0.001 are presented in Table 8 for all four deblurring methods and the original method. Once again, all four deblurring methods are proven to be beneficial for matching heterogeneous images with different quality, with our SVD-based deblurring network substantially outperforming the other five deblurring methods. GAR and EER are improved from 92.23% and 8.71% to 96.08% and 5.59%, and from 64.48% and 23.24% to 73.80% and 18.53% for NIR 50 m and 106 m, respectively. Again, it can be concluded that our proposed method of heterogeneous quality balancing is beneficial for cross-spectral face recognition and is superior to other deblurring methods, including state of the art. Such a statement is especially true for longer distances of image acquisition.

### 4.5. Analysis and Conclusion

In this subsection, we would like to reiterate and summarize a number of conclusions made earlier in the text regarding the benefit of using the quality balancing approach proposed in this paper.

- Infrared faces acquired at long standoffs suffer from quality degradation due to atmospheric and camera effects, which leads to a serious drop in the cross-spectral recognition performance, raising the issue of heterogeneous image quality imbalance.
- For both SWIR and NIR at 50 m and 106 m, image quality balancing prior to face matching via upgrading the low quality imagery (i.e., cascaded enhancement) or downgrading the high quality imagery (Gaussian smoothing) yields substantial improvement in recognition performance, with the former approach being better than the latter approach.
- The proposed cascaded enhancement structure is necessary and effective in that a single denoising stage yields lower recognition performance, while a subsequent deblurring stage dramatically improves the performance.
- In the context of cross-spectral face recognition, the newly developed deblurring network (SVDFace) demonstrates its advantage over traditional deblurring methods, as well as the state-of-the-art deblurring model based on deep learning, for all cases of IR bands and standoffs.
- As the degree of quality imbalance between the heterogeneous faces increases, such as when the standoff increases from 50 m to 106 m, the effect of quality balancing becomes more pronounced, especially for the SWIR band.

## 5. Conclusions

This paper studies the issue of heterogeneous quality imbalance in cross-spectral face recognition. To compensate the quality disparity between the heterogeneous imageries, we propose upgrading the quality of IR faces via a cascaded structure of face enhancement, the core of which is featured by a SVD theory-inspired deblurring deep neural networks.

Our proposed approach of quality balancing is tested on a dataset composed of heterogeneous face images acquired in visible light, NIR and SWIR. To demonstrate the advantage of the proposed approach, we conduct cross-spectral face recognition experiments and compare our method against the others. Firstly, in all considered cases, the proposed method of cascaded enhancement yields significantly improved performance than the original method without any preprocessing of quality balancing at all. Secondly, our method is also superior to the downgrading method via Gaussian smoothing, and the cascaded structure is better than the non-cascaded structure. Lastly, the proposed SVD deblurring model (SVDFace) outperforms five other deblurring methods including state of the art. This is especially pronounced at a longer standoff.

## Figures and Tables

**Table 1:** Parameters of the proposed deep neural network of deblurring.

Index	Type	Patch Size	Remark	Output Size
1	C1_1	5 × 1	vertical	116 × 112 × 16
2	C1_2	5 × 1	vertical	112 × 112 × 16
3	C1_3	5 × 1	vertical	108 × 112 × 16
4	C1_4	5 × 1	vertical	104 × 112 × 16
5	C1_5	5 × 1	vertical	100 × 112 × 16
6	C1_6	5 × 1	vertical	96 × 112 × 16
7	C1_7	5 × 1	vertical	92 × 112 × 16
8	C1_8	5 × 1	vertical	88 × 112 × 16
9	C1_9	5 × 1	vertical	84 × 112 × 16
10	C1_10	5 × 1	vertical	80 × 112 × 16
11	C1_11	5 × 1	vertical	76 × 112 × 16
12	C2_1	1 × 5	horizontal	76 × 108 × 16
13	C2_2	1 × 5	horizontal	76 × 104 × 16
14	C2_3	1 × 5	horizontal	76 × 100 × 16
15	C2_4	1 × 5	horizontal	76 × 96 × 16
16	C2_5	1 × 5	horizontal	76 × 92 × 16
17	C2_6	1 × 5	horizontal	76 × 88 × 16
18	C2_7	1 × 5	horizontal	76 × 84 × 16
19	C2_8	1 × 5	horizontal	76 × 80 × 16
20	C2_9	1 × 5	horizontal	76 × 76 × 16
21	C2_10	1 × 5	horizontal	76 × 72 × 16
22	C3_1	5 × 5	square	72 × 68 × 32
23	C3_2	5 × 5	square	68 × 64 × 32
24	C3_3	5 × 5	square	64 × 60 × 32
25	C3_4	5 × 5	square	60 × 56 × 32
26	C4_1	1 × 1	up-sampling	120 × 112 × 128
27	C4_2	5 × 5	padding	120 × 112 × 1

**Table 2:** Advantages of SVD-inspired networks over other networks of ordinary convolutions. Assume an input image of m×n and a SVD networks with *l* layers, where each layer has *k* kernels.

Network Type	Dimensions	Cost	Numbers of Parameters	Training Data Needed	Interpretable
Vertical SVD Networks	1D	Low	3×m×n×l×k	Small	Yes
Horizontal SVD Networks	1D	Low	3×m×n×l×k	Small	Yes
Other Networks	2D	High	9×m×n×l×k	Large	No

**Table 3:** Equal Error Rate (EER) and Genuine Accept Rates (GARs) at False Accept Rate (FAR) = $10^{-1}$ (GAR1) and FAR = $10^{-3}$ (GAR2) for the cases of SWIR 50 m and 106 m: The upgrading approach vs. the downgrading approach.

CASE	METHOD	GAR1 (%)	GAR2 (%)	EER (%)
SWIR 50 m	Original	91.88	62.11	8.90
	Downgrading (Gaussian Smoothing)	92.93	67.09	7.92
	Upgrading (proposed)	96.43	69.26	5.53
SWIR 106 m	Original	82.50	44.79	14.17
	Downgrading (Gaussian Smoothing)	86.74	51.67	11.75
	Upgrading (proposed)	91.8	52.78	9.04

**Table 4:** EER and GARs at FAR = $10^{-1}$ (GAR1) and FAR = $10^{-3}$ (GAR2) for the cases of NIR 50 m and 106 m: The upgrading approach vs. the downgrading approach.

CASE	METHOD	GAR1 (%)	GAR2 (%)	EER (%)
NIR 50 m	Original	92.23	68.21	8.71
	Downgrading (Gaussian Smoothing)	93.42	70.24	7.63
	Upgrading (proposed)	96.08	70.45	5.95
NIR 106 m	Original	64.48	13.28	23.24
	Downgrading (Gaussian Smoothing)	66.38	15.96	21.73
	Upgrading (proposed)	73.80	17.79	18.53

**Table 5:** EER and GARs at FAR = $10^{-1}$ (GAR1) and FAR = $10^{-3}$ (GAR2) for the cases of SWIR 50 m and 106 m: cascaded vs. non-cascaded.

CASE	METHOD	GAR1 (%)	GAR2 (%)	EER (%)
SWIR 50 m	Original	91.88	62.11	8.90
	Non-cascaded (BM3D alone)	88.23	53.92	11.21
	Cascaded Enhancement (BM3D + CNN deblur)	96.43	69.26	5.53
SWIR 106 m	Original	82.50	44.79	14.17
	Non-cascaded (BM3D alone)	74.79	35.56	18.33
	Cascaded enhancement (BM3D + CNN deblur)	91.81	52.78	9.04

**Table 6:** EER and GARs at FAR = $10^{-1}$ (GAR1) and FAR = $10^{-3}$ (GAR2) for the cases of NIR 50 m and 106 m: cascaded vs. non-cascaded.

CASE	METHOD	GAR1 (%)	GAR2 (%)	EER (%)
NIR 50 m	Original	92.23	68.21	8.71
	Non-cascaded (BM3D alone)	91.04	65.97	9.38
	Cascaded enhancement (BM3D + CNN deblur)	96.08	70.45	5.95
NIR 106 m	Original	64.48	13.28	23.24
	Non-cascaded (BM3D alone)	60.95	8.97	23.37
	Cascaded enhancement (BM3D + CNN deblur)	73.80	17.79	18.53

**Table 7:** EER and GARs at FAR = $10^{-1}$ (GAR1) and FAR = $10^{-3}$ (GAR2): Matching SWIR 50 m and 106 m probes to visible gallery with different deblurring methods. The method of the best performance is shown in bold.

CASE	METHOD	GAR1 (%)	GAR2 (%)	EER (%)
SWIR 50 m	Original	91.88	62.11	8.90
	Laplacian Sharpening [22]	94.33	67.44	7.29
	Blind Deconvolution [47]	92.99	65.90	8.04
	DeblurGan [42]	93.56	63.10	7.59
	Progressive Semantic Deblurring [48]	93.28	62.54	7.85
	UMSN Face Deblurring [49]	94.05	61.55	7.28
	**SVDFace** (proposed)	**96.43**	**69.26**	**5.53**
SWIR 106 m	Original	82.50	44.79	14.17
	Laplacian Sharpening [22]	90.00	52.15	10.00
	Blind Deconvolution [47]	84.65	47.85	13.42
	DeblurGan [42]	89.80	47.91	10.14
	Progressive Semantic Deblurring [48]	87.15	43.13	11.67
	UMSN Face Deblurring [49]	88.40	46.88	10.97
	**SVDFace** (proposed)	**91.81**	**52.78**	**9.04**

**Table 8:** EER and GARs at FAR = $10^{-1}$ (GAR1) and FAR = $10^{-3}$ (GAR2): Matching NIR 50 m and 106 m probes to visible gallery with different deblurring methods. The method of the best performance is shown in bold.

CASE	METHOD	GAR1 (%)	GAR2 (%)	EER (%)
NIR 50 m	Original	92.23	68.21	8.71
	Laplacian Sharpening [22]	94.12	70.87	7.12
	Blind Deconvolution [47]	92.23	69.26	8.47
	DeblurGan [42]	93.28	65.97	8.19
	Progressive Semantic Deblurring [48]	92.44	64.71	8.53
	UMSN Face Deblurring [49]	93.27	65.90	7.99
	**SVDFace** (proposed)	**96.08**	**70.45**	**5.95**
NIR 106 m	Original	64.48	13.28	23.24
	Laplacian Sharpening [22]	66.81	16.38	20.39
	Blind Deconvolution [47]	63.91	17.58	21.29
	DeblurGan [42]	65.75	15.40	21.32
	Progressive Semantic Deblurring [48]	64.62	15.25	21.45
	UMSN Face Deblurring [49]	66.60	15.54	20.67
	**SVDFace** (proposed)	**73.80**	**17.79**	**18.53**

## Data Availability

No new data were created or analyzed in this study. Data sharing is not applicable to this article.
